# Cell-Type-Selective Effects of Intramembrane Cavitation as a Unifying Theoretical Framework for Ultrasonic Neuromodulation^1^,^2^,^3^

**DOI:** 10.1523/ENEURO.0136-15.2016

**Published:** 2016-06-22

**Authors:** Michael Plaksin, Eitan Kimmel, Shy Shoham

**Affiliations:** Faculty of Biomedical Engineering and Russell Berrie Nanotechnology Institute, Technion-Israel Institute of Technology, Haifa 32000, Israel

**Keywords:** action potential, Hodgkin and Huxley, model, neurons, T-type calcium channels, ultrasound

## Abstract

Diverse translational and research applications could benefit from the noninvasive ability to reversibly modulate (excite or suppress) CNS activity using ultrasound pulses, however, without clarifying the underlying mechanism, advanced design-based ultrasonic neuromodulation remains elusive. Recently, intramembrane cavitation within the bilayer membrane was proposed to underlie both the biomechanics and the biophysics of acoustic bio-effects, potentially explaining cortical stimulation results through a neuronal intramembrane cavitation excitation (NICE) model. Here, NICE theory is shown to provide a detailed predictive explanation for the ability of ultrasonic (US) pulses to also suppress neural circuits through cell-type-selective mechanisms: according to the predicted mechanism T-type calcium channels boost charge accumulation between short US pulses selectively in low threshold spiking interneurons, promoting net cortical network inhibition. The theoretical results fit and clarify a wide array of earlier empirical observations in both the cortex and thalamus regarding the dependence of ultrasonic neuromodulation outcomes (excitation-suppression) on stimulation and network parameters. These results further support a unifying hypothesis for ultrasonic neuromodulation, highlighting the potential of advanced waveform design for obtaining cell-type-selective network control.

## Significance Statement

Recent studies have demonstrated that ultrasound waves are capable of stimulating or suppressing neural circuits, thereby opening up an important new route toward targeted noninvasive neuromodulation. However, the underlying mechanism for ultrasonically eliciting specific neuromodulatory effects has not been clarified. Our new theoretical analysis reveals that ultrasound can selectively excite different cortical neuron subtypes simply by changing the stimulation pattern, driven by the response properties of T-type calcium channels. Interestingly, the model’s predictions at the single-neuron and network levels are shown to closely agree with and explain the emerging field’s entire body of experimental results, spanning from rodents to humans, and can thus facilitate the development of new ways of treating or diagnosing brain disorders.

## Introduction

Both classical and recently emerging data have demonstrated that the interaction of therapeutic ultrasound (US) and excitable tissues leads to a diverse and complex set of reversible physiological phenomena which are collectively referred to as ultrasonic neuromodulation (Bystritsky et al., 2011; Yoo et al., 2011a; Kim et al., 2015; Naor et al., 2016). US-induced neuromodulation phenomena include the generation of action potentials (APs) in CNS neurons in the brain (Tyler et al., 2008; Tufail et al., 2010, 2011; Kim et al., 2012, 2014; King et al., 2013, 2014) and the retina (Naor et al., 2012; Menz et al., 2013), suppression of CNS activity (Yoo et al., 2011a; Min et al., 2011; Kim et al., 2015) and of nerve conduction (Young and Henneman, 1961; Lele, 1963; Colucci et al., 2009), as well as more subtle changes in excitability (Moore et al., 2000; Tsui et al., 2005). The development of ultrasonic neuromodulation is largely motivated by future therapeutic applications. This is the only neuromodulation technology currently capable of being selective, targeted, reversible and noninvasive with millimeter-scale precision essentially across the entire human and nonhuman primate brain (Deffieux et al., 2013; Coluccia et al., 2014; Legon et al., 2014; Lee et al., 2015) and peripheral nervous system (Tsui et al., 2005; Colucci et al., 2009; Juan et al., 2014). Although ultrasonic neuromodulation phenomena could potentially result from a diverse set of different biophysical interaction modes, including temperature elevation (Lele, 1963; Colucci et al., 2009), acoustic streaming (Tyler, 2011), radiation pressure (Tyler, 2011; Prieto et al., 2013), and stable or inertial cavitation (Tyler, 2011), only the recently proposed intramembrane cavitation hypothesis (Krasovitski et al., 2011) and the related neuronal intramembrane cavitation excitation (NICE) model (Plaksin et al., 2014) have led to a detailed predictive explanation of specific empirical results for CNS stimulation by low-intensity US. In the NICE framework, US-induced oscillatory expansions and contractions of small membrane regions, the bilayer sonophores (BLSs; Krasovitski et al., 2011), cause capacitive displacement currents that indirectly lead to slow membrane charge accumulation (on a time scale of tens of milliseconds), ultimately bringing the neurons to their AP discharge threshold. The NICE model explains the basic features of acoustic cortical neuro-stimulation of pyramidal neurons, including the long durations required and the apparent higher efficiency of continuous wave (CW) versus pulse-mode (discontinuous) stimulation (Tufail et al., 2010, 2011; King et al., 2013), and its predictions have a very good agreement with the recent experimental results of detailed parameter-dependent efficacy for mouse motor cortex direct ultrasonic stimulation (King et al., 2013). In contrast, the biophysical basis of CNS neural suppression (Yoo et al., 2011a; Kim et al., 2015) remains elusive, and the ability to differentially mediate suppression simply by varying the stimulation parameters, did not receive a rigorous, quantitative, and predictive treatment. Fundamentally, it is unclear whether suppression is mediated by directly suppressing active excitatory neurons, by activating inhibitory neurons, or by depleting excitatory synaptic pools, to mention just a few possibilities.

Here, we extend the emerging intramembrane cavitation framework to examine the predicted effects of US on additional cell types and stimulation modes. We analyze the effect of pulsed mode US with parameters ranging from CW to low duty-cycle (previously shown to suppress cortical and thalamic activity; Yoo et al., 2011a; Min et al., 2011; Kim et al., 2015) on NICE-type models of multiple types of cortical neurons and related thalamic neurons. The new analysis reveals that low-threshold spiking (LTS) inhibitory cortical neurons (Pospischil et al., 2008) and major types of thalamic neurons (Destexhe et al., 1998a) are hyper-sensitive during discontinuous pulsed US stimulation. In these neurons, the presence of T-type voltage-gated calcium channels boosts charge accumulation between the short US bursts, leading to their selective activation when low duty-cycle waveforms are applied. These differential US-induced responses of different cell types are shown to explain the emergence of neuromodulation parameter ranges for obtaining network stimulation or suppression that match and clarify empirical parameter choices. Moreover, how these effective parameter ranges are modulated by baseline input into the cortical network provide further empirical predictions regarding the dependence on thalamic input, anesthetic modulation, etc.

## Theoretical framework

### The biomechanical-biophysical models

**Figure 1.**
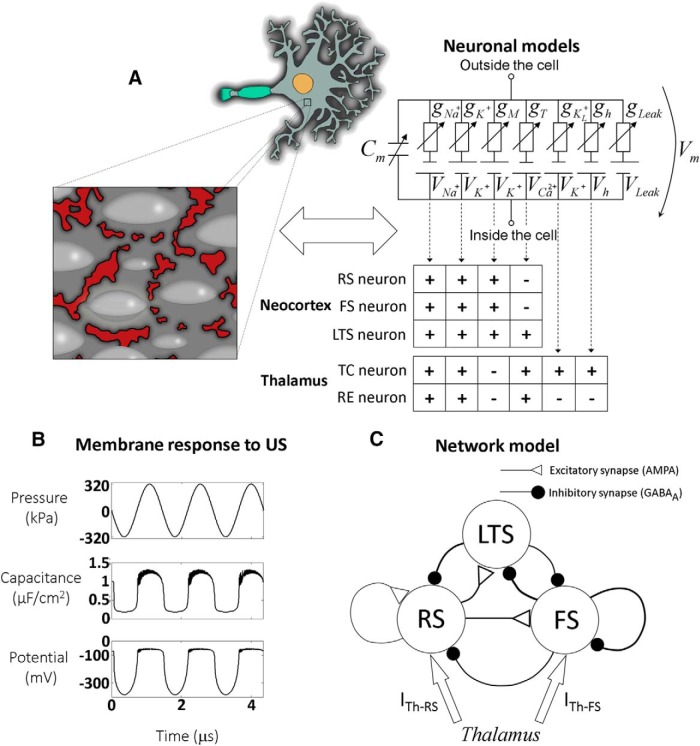
Cortical and thalamic NICE models. ***A***, Geometrical and biophysical representation structure of the NICE models: top view (left) of the US-induced dome-shaped BLS intermembrane cavities (light gray) in the plasma membrane bare zones (dark grey), bounded by cholesterol-rich protein islands (red areas). The equivalent electrical circuit of this biophysical complex structure (right) includes a potential (*V*_m_), time-varying capacitance (*C*_m_), and Hodgkin–Huxley type ionic conductances (*g*_i_) and sources (*V*_i_). Each neuron type channels' composition is summarized in the neocortical and thalamic tables. ***B***, Electrical dynamics during first three cycles of the model membrane exposed to US (*f*=0.69 MHz, 3.3 W/cm^2^): acoustic pressure (kPa), membrane capacitance (μF/cm^2^), and membrane potential (mV). ***C***, A simplified network of RS, FS, and LTS cortical neurons. The filled black circles and open triangles are GABA_A_ and AMPA- type synapses, respectively. The excitatory connections to the two FS and LTS inhibitory neurons are depressing and facilitating, respectively. The synaptic strength is represented by changes of the lines' thickness (logarithmically scaled) and *I_Th-RS_* and *I_Th-FS_* are the thalamic inputs.

We simulated and analyzed the expected effect of US on five types of mammalian cortical and thalamic cell types where detailed BLS-type membrane interactions are coupled with Hodgkin-Huxley (H&H) single-compartment models of three cortical neuron types (Pospischil et al., 2008) and two central types of thalamic neurons (Destexhe et al., 1998a). The cortical neuron types include an excitatory regular spiking (RS) pyramidal neuron and two principal models for cortical inhibitory interneurons: LTS and fast spiking (FS) neurons, whereas the thalamic types included thalamocortical (TC) and thalamic reticular (RE) neurons. The various NICE model parameters were taken “as is” without retuning or *post hoc* adjustments, and are generally based on previously measured or estimated physical and biophysical quantities (summarized in Table 1 with the respective sources). In the model, a circular, uniform phospholipid bilayer membrane dome is bounded between immobile protein islands (Fig. 1*A*; Plaksin et al.,2014); structured intramembrane cavities appear to be consistent with the observed clustering of protein distributions in real cells’ membranes (Lillemeier et al., 2006, their Fig. 1, where protein-free patches of 50–100 nm diameters are evident). Electrically, the bilayer membrane has a varying capacitance, and each ion has a Nernst equilibrium potential and a time-dependent conductance (Fig. 1*A*, right), which generally depends on the product probabilities of multiple voltage-dependent gates (M and H gates for sodium channels; N and P gates for potassium channels and S and U for calcium channels; Pospischil et al., 2008). When US is applied, the membrane leaflets oscillate according to modified Rayleigh–Plesset bubble dynamics (Krasovitski et al., 2011), causing oscillatory variations in the membrane capacitance (Fig. 1*B*), which lead to an oscillatory displacement current *V_m_dC_m_/dt* that directly modulates the membrane potential (Plaksin et al., 2014). The responses of the nanometer-scale BLS model to US are assumed to be representative of the responses of the whole cell; US waves with sub-millihertz frequencies have wavelengths of several millimeters, orders of magnitude larger than the dimensions of CNS cortical neuron somata, so all BLS elements are subject to essentially the same acoustic effect (Plaksin et al., 2014).

**Table 1.** Biomechanical and biophysical parameters for the simulation runs

	**Parameter**	**Symbol**	**Unit**	**Value**	**Source**
**Biomechanical Parameters**					
1	Thickness of the leaflet	$\delta_{0}$	nm	2	Plaksin et al., 2014
2	Initial gap between the two leaflets (uncharged)	$\Delta^{*}$		1.4	
3	Initial gap between the two leaflets (when charged)	Δ		1.26 (RS)	Calculated from equilibrium state using Plaksin et al., 2014, their Eq. 2
4				1.26 (FS)	
5				1.3 (LTS)	
6				1.28 (TC)	
7				1.21 (RE)	
8	Attraction/repulsion pressure coefficient	$A_{r}$	Pa	10^5^	Plaksin et al., 2014
9	Exponent in the repulsion term	*x*	—	5	
10	Exponent in the attraction term	*_y_*	—	3.3	
11	Dynamic viscosity of the leaflets	$\mu_{s}$	Pa·s	0.035	
12	Dynamic viscosity of the surrounding medium	$\mu_{I}$		0.7·10^−3^	
13	Diffusion coefficient of air in the surrounding medium	$D_{a}$	m^2^·s^−1^	3·10^−9^	
14	Density of the surrounding medium	$\rho_{l}$	kg·m^−3^	1028	
15	Speed of sound in the surrounding medium	c	m·s^−1^	1515	
16	Initial air molar concentration in the surrounding medium (O_2_+N_2_)	$C_{a}$	mol·m^−3^	0.62	
17	Henry’s constant for dissolved air in the surrounding medium	$k_{a}$	Pa·m^3^·mol^−1^	1.63·10^5^	
18	Static pressure in the surrounding medium	$P_{0}$	Pa	10^5^	
19	Radius of the leaflets' boundary	*a*	nm	32	
20	Width of the boundary layer between the surrounding medium and the leaflets	ξ		0.5	
21	Areal modulus of the bilayer membrane	$k_{s}$	N·m^−1^	0.24	
22	Relative permittivity of the intramembrane cavity	$\varepsilon_{r}$	—	1	
23	Membrane baseline capacitance per unit area	$C_{m_{0}}$	µF·cm^−2^	1	
24	Surrounding medium temperature	*Tem*	K	309.15	Pospischil et al., 2008; Destexhe et al., 1998a
**Biophysical parameters**					
25	Maximal conductance of Na^+^ channels	G¯+Na	mS·cm^−2^	56 (RS)	Pospischil et al., 2008
				50 (RS; Fig. 7)	
26				58 (FS)	
				50 (FS; Fig. 7)	
27				50 (LTS)	
28				90 (TC)	Destexhe et al., 1998a
29				200 (RE)	
30	Maximal conductance of delayed-rectiﬁer K^+^ channels	G¯+K		6 (RS)	Pospischil et al., 2008
				5 (RS; Fig. 7)	
31				3.9 (FS)	
				10 (FS; Fig. 7)	
32				4 (LTS)	
				5 (LTS; Fig. 7)	
33				10 (TC)	Destexhe et al., 1998a
34				20 (RE)	
35	Maximal conductance of slow non-inactivating K^+^ channels	${\bar{G}}_{M}$		0.075 (RS)	Pospischil et al., 2008
				0.07 (RS; Fig. 7)	
36				0.0787 (FS)	
				0 (FS; Fig. 7)	
37				0.028 (LTS)	
				0.03 (LTS; Fig. 7)	
38	Maximal conductance of low-threshold Ca^2+^ channels	${\bar{G}}_{T}$		0.4 (LTS)	
39				2 (TC)	Destexhe et al., 1998a
40	Maximal conductance of low- threshold Ca^2+^ channels	${\bar{G}}_{T_{s}}$		3 (RE)	
41	Maximal conductance of leak potassium currents	${\overset{¯}{G}}_{K_{L}^{+}}$		0.0138 (TC)	
42	Maximal conductance of hyperpolarization-activated mixed cationic current	${\bar{G}}_{h}$		0.0175 (TC)	
43	Maximal conductance of non-voltage-dependent, nonspecific ions channels	${\bar{G}}_{Leak}$		0.0205 (RS)	Pospischil et al., 2008
				0.1 (RS; Fig. 7)	
44				0.038 (FS)	
				0.15 (FS; Fig. 7)	
45				0.019 (LTS)	
				0.01 (LTS; Fig. 7)	
46				0.01 (TC)	Destexhe et al., 1998a
47				0.05 (RE)	
48	Nernst potential of Na^+^	V+Na	mV	50	Pospischil et al., 2008
49	Nernst potential of K^+^	V+K		−90	
50	Nernst potential of Ca^2+^ (LTS neuron)	V2+Ca		120	
51	Reversal potential of a hyperpolarization-activated mixed cationic current	$V_{h}$		−40	Destexhe et al., 1996a
52	Nernst potential of non-voltage-dependent, nonspecific ion channels	$V_{Leak}$		−70.3 (RS)	Pospischil et al., 2008
				−70 (RS; Fig. 7)	
53				−70.4 (FS)	
				−70 (FS; Fig. 7)	
54				−50 (LTS)	
				−85 (LTS; Fig. 7)	
55				−70 (TC)	Destexhe et al., 1998a
56				−90 (RE)	
57	Spike threshold adjustment parameter	$V_{T}$		−56.2 (RS)	Pospischil et al., 2008
				−55 (RS; Fig. 7)	
58				−57.9 (FS)	
				−55 (FS; Fig. 7)	
59				−50 (LTS)	
				−55 (LTS; Fig. 7)	
60				−52 (TC)	Destexhe et al., 1998b
61				−67 (RE)	Destexhe et al., 1996b
62	Decay time constant for adaptation at slow non-inactivating K^+^ channels	$\tau_{\max}$	ms	608 (RS)	Pospischil et al., 2008
				1000 (RS; Fig. 7)	
63				502 (FS)	
				1000 (FS; Fig. 7)	
64				4000 (LTS)	
				1000 (LTS; Fig. 7)	
65	The resting potential of the cell membrane	$V_{m_{0}}$	mV	−71.9 (RS)	Calculated from Pospischil et al., 2008
				−70.4 (RS; Fig. 7)	
66				−71.4 (FS)	
				−70 (FS; Fig. 7)	
67				−54 (LTS)	
				−84.6 (LTS – Fig. 7)	
68				−63.4 (TC)	Calculated from Destexhe et al., 1998a
69				−89.5 (RE)	
70	The effective depth beneath the membrane area for calcium concentration calculations (for TC and RE neurons)	d	nm	100	Destexhe et al., 1998a and Destexhe et al., 1996a
71	An extracellular Ca^2+^ concentration (for TC and RE neurons)	${Ca}_{o}^{2+}$	mm	2	
72	Decay time constants of Ca^2+^ (for TC and RE neurons)	τ2+Ca	ms	5	
73	$I_{h}$ current Ca^2+^ regulation factor	$k_{1}$	mm ^−4^· ms^−1^	2.5·10^7^	
74	$I_{h}$ current Ca^2+^ regulation factor	$k_{2}$	ms^−1^	4·10^−4^	
75	$I_{h}$ current Ca^2+^ regulation factor	$k_{3}$		0.1	
76	$I_{h}$ current Ca^2+^ regulation factor	$k_{4}$		0.001	
77	FS to RS neuron thalamic input current ratio	*R_TH_*	—	1.4	Hayut et al., 2011
78	Thalamic DC current input to the RS neuron	$I_{Th-RS}$	nA	0.17	Based on Destexhe and Paré.,1999
79	AMPA synaptic currents reversal potential	$V_{AMPA}$	mV	0	Destexhe et al., 1996a
80	GABA_A_ synaptic currents reversal potential	$V_{GABA_{A}}$		-85	
81	Total maximal synaptic conductance used for RS to RS connection	${\bar{g}}_{RS-RS}$	μS	0.002	Calculated from Vierling-Claassen et al., 2010
82	Total maximal synaptic conductance used for RS to FS connection	${\bar{g}}_{RS-FS}$		0.04	
83	Total maximal synaptic conductance used for RS to LTS connection	${\bar{g}}_{RS-LTS}$		0.09	
84	Total maximal synaptic conductance used for FS to RS connection	${\bar{g}}_{FS-RS}$		0.015	
85	Total maximal synaptic conductance used for FS to FS connection	${\bar{g}}_{FS-FS}$		0.135	
86	Total maximal synaptic conductance used for FS to LTS connection	${\bar{g}}_{FS-LTS}$		0.86	
87	Total maximal synaptic conductance used for LTS to RS connection	${\bar{g}}_{LTS-RS}$		0.135	
88	Total maximal synaptic conductance used for LTS to FS connection	${\bar{g}}_{LTS-FS}$		0.02	
89	AMPA rise time constant	$t_{1}$	ms	0.1	Vierling-Claassen et al., 2010
90	AMPA decay time constant	$t_{2}$		3	
91	GABA_A_ rise time constant from FS neuron	$t_{1}$		0.5	
92	GABA_A_ decay time constant from FS neuron	$t_{2}$		8	
93	GABA_A_ rise time constant from LTS neuron	$t_{1}$		0.5	
94	GABA_A_ decay time constant from LTS neuron	$t_{2}$		50	
95	Short-term synaptic plasticity facilitation factor (from RS to LTS)	*f*	—	0.2	
96	Short-term synaptic plasticity facilitation factor time constant (from RS to LTS)	$\tau_{f}$	ms	200	
97	Short-term synaptic plasticity facilitation factor (from RS to FS)	*f*	—	0.5	
98	Short-term synaptic plasticity facilitation factor time constant (from RS to FS)	$\tau_{f}$	ms	94	
99	Short-term synaptic plasticity short-time depression factor (from RS to FS)	$d_{1}$	—	0.46	
100	Short-term synaptic plasticity short-time depression factor time constant (from RS to FS)	$\tau_{d_{1}}$	ms	380	
101	Short-term synaptic plasticity long-time depression factor (from RS to FS)	$d_{2}$	—	0.975	
102	Short-term synaptic plasticity long-time depression factor time constant (from RS to FS)	$\tau_{d_{2}}$	ms	9200	
103	Neuronal cell membrane area	*A *	μm^2^	11.88·10^3^ (RS)	Pospischil et al., 2008
104				10.17·10^3^ (FS)	
105				25·10^3^ (LTS)	
106				29·10^3^ (TC)	Destexhe et al., 1998a
107				14·10^3^ (RE)	

The synaptic strengths were calculated from Vierling-Claassen et al. (2010), multiplying their individual synaptic strengths by the average number of converging connections from each type (Vierling-Claassen et al., 2010, their Table 3) and by the ratio of membrane areas between the NICE-neuron model and the respective model in their study. The latter normalization is consistent with an assumption that the total number of putative synapses on the dendrites and soma are proportional to a neuron's size (Gibbins et al., 1998).

To explore the significance of network interactions and baseline activity on acoustic neuromodulation we explored the effect of US stimuli on a spiking cortical network model with thalamic input (Fig. 1*C*; Hayut et al., 2011) where the RS, FS, and LTS cell types are coupled through excitatory (AMPA) and inhibitory (GABA_A_) synaptic connections whose strength and short-term dynamics are based on Vierling-Claassen et al. (2010).

### Models' equations

The governing mathematical expression used in our study are H&H-based single compartment model equations, adapted to mammalian RS, FS, and LTS cortical neurons (Pospischil et al., 2008) and thalamic TC and RE neurons (Destexhe et al., 1998a); in addition to the H&H ionic currents, the models include US-induced displacement currents (Plaksin et al., 2014). Excitatory and inhibitory synaptic currents (Vierling-Claassen et al., 2010) and thalamic inputs to the RS and FS neurons (but not to the LTS) were added to the neocortical network simulations (Hayut et al., 2011):

CmdVmdt+VmdCmdt=−I+Na−I+K−IM−IT/Ts−Ih−−IKL+−ILeak−∑iIGABAA−IAMPA−ITh,

where $V_{m}$ is the cell membrane potential; $C_{m}$ is the cell membrane capacitance; $\frac{dC_{m}}{dt}V_{m}$ represents the capacitive displacement current induced by the US-subjected BLS dynamics, leading to changes in the average membrane capacitance (Plaksin et al., 2014; Fig. 1*B*); I+Na, I+K, $I_{M}$, $I_{T/T_{s}}$, $I_{h}$, $I_{K_{L}^{+}}$ and *I_Leak_* are the currents of the sodium, delayed-rectiﬁer potassium, slow non-inactivating potassium, low-threshold calcium, hyperpolarization-activated mixed cationic, leak potassium and the non-voltage-dependent nonspecific ion channels, respectively [I+Na, I+K, and $I_{Leak}$ exists in all NICE models and their dynamics were taken from Pospischil et al. (2008); $I_{T_{s}}$ only in the LTS and TC neurons, Huguenard and McCormick (1992); $I_{T_{s}}$ only in the RE neuron, Destexhe et al. (1996b); $I_{h}$ and $I_{K_{L}^{+}}$ only in the TC neuron, Destexhe et al. (1996a) and $I_{M}$ only in the RS, FS, and LTS neurons, Pospischil et al., (2008)]; $\sum_{i}I_{GABA_{A}}$ is the sum of FS and LTS neurons related GABA_A_ synapses induced inhibitory currents; $I_{AMPA}$ is the RS neuron related AMPA synapses induced excitatory current and $I_{Th}$ is the thalamic DC current input to the RS and FS neurons with *I_Th-FS_/I_Th-RS_*=1.4, following Hayut et al. (2011; see also Cruikshank et al., 2007), and *I_Th-RS_* =0.17 nA, which generates baseline cortical pyramidal neuron average firing rate of about 7Hz, matching the average spontaneous firing rate of ketamine-xylazine anesthetized animals (Destexhe and Paré., 1999).

The network model structure was adopted from Hayut et al. (2011), with synaptic currents modeled as in Vierling-Claassen et al. (2010):

$$I_{GABA_{A}/AMPA}={\bar{g}}_{ij}P(t)s(t)(V_{m}-V_{ij}),$$

where *i* and *j* indexes can be RS, FS, and LTS related to the presynaptic and postsynaptic neurons, respectively; P(t) relates to the short term synaptic plasticity for the excitatory connections to both the inhibitory neurons as described by Vierling-Claassen et al. (2010) and modeled as by Varela et al. (1997); s(t) relates to synaptic open probability modeled as bi-exponential function (Kleppe and Robinson, 1999) and $V_{ij}$ and ${\bar{g}}_{ij}$ represents the AMPA or GABA_A_ synaptic currents reversal potentials and strengths, respectively.

All the models' biomechanical, biophysical, and synaptic parameters and their respective sources can be found in Table 1.

### LTS and TC neurons T-type calcium channels current dynamics

The LTS inhibitory interneuron's T-type calcium channels current was modeled as by Pospischil et al. (2008):

$$\begin{array}{l} I_{T}={\bar{G}}_{T}s^{2}u(V_{m}-V_{Ca})\\ \frac{dx}{dt}=(x_{\infty }(V_{m})-x)/\tau_{x}(V_{m}), \end{array}$$

where *V_Ca_* is the Nernst potential of the calcium ions; *s* and *u*, designate the open probabilities of the activation and inactivation S-type and U-type gates, respectively, and x, $x_{\infty }$, and $\tau_{x}(V_{m})$ are the open probability, voltage-dependent steady state open probability and voltage-dependent time constant of the S-type or U-type gates, respectively.

The gates' dynamics are based on Huguenard and McCormick (1992):

$$\begin{array}{l} s_{\infty }(V_{m})=\frac{1}{1+e^{-(V_{m}+V_{x}+57)/6.2}}\\ u_{\infty }(V_{m})=\frac{1}{1+e^{(V_{m}+V_{x}+81)/4}} \end{array}$$

$$\begin{array}{c} \tau_{s}(V_{m})=\frac{1}{3.7}\left[0.612+\frac{1}{e^{-\left(\frac{V_{m}+V_{x}+132}{16.7}\right)}+e^{\left(\frac{V_{m}+V_{x}+16.8}{18.2}\right)}}\right]\\ \tau_{u}(V_{m})=\left\{ \begin{array}{l} (V_{m}+V_{x})<-80\;mV\;\frac{1}{3.7}e^{\left(\frac{V_{m}+V_{x}+467}{66.6}\right)}\\ (V_{m}+V_{x})\geq -80\;mV\;\frac{1}{3.7}\left[{e^{-}}^{\left(\frac{V_{m}+V_{x}+22}{10.5}\right)}+28\right] \end{array}\right. \end{array},$$

where the time constants were adapted from of 24^°^C to 36^°^C, using a Q_10_ factor of 3 (Pospischil et al., 2008) and *V*_x_=−7 mV (*V*_x_=−2 mV for Fig. 7) is a voltage-dependence uniform shift factor defined by Pospischil et al. (2008). The TC neuron's T-type calcium channels gates’ dynamics are also described by these expressions, with *V*_x_=0 mV (Huguenard and McCormick, 1992; Destexhe et al., 1998a).

### Models’ implementation

The model’s set of equations was numerically solved in MATLAB (using the function ODE113). The time difference between the calculated points was set to 0.025/f µs (where *f* is the US frequency in megahertz). The NICE models, including the network model, were solved in the same manner as explained in our previous study (Plaksin et al., 2014).

The US intensities (*I*) reported in this study were calculated in the form of spatial peak-pulse average intensities for propagating planar US waves (Hendee and Ritenour, 2002):

$$I=\frac{P_{A}^{2}}{2\rho_{l}c},$$

where *P_A_* is the pressure amplitude, $\rho_{l}$ is the surrounding medium density, and *c* is the speed of sound in the medium.

## Results

### Prediction I: the responses of cortical neurons to ultrasound are strongly cell-type and waveform-dependent

**Figure 2.**
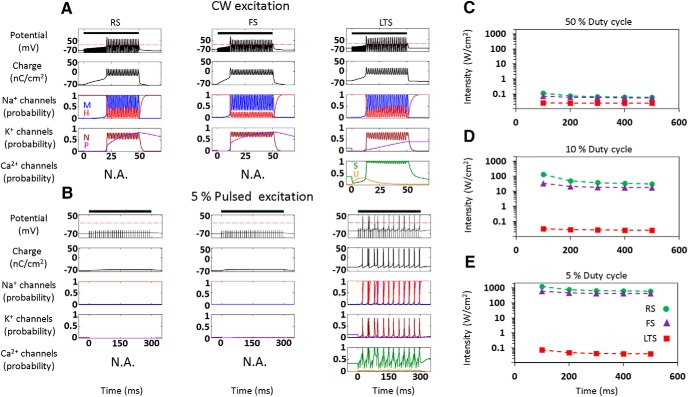
Effect of continuous and pulsed US stimuli on the different cortical NICE-neurons (*f*=0.69 MHz). ***A***, ***B***, Effect of US stimulus (3.3 W/cm^2^, indicated by bars) on membrane potential and charge (top), sodium and potassium channels kinetics (middle), and on LTS neuron T-type calcium channels kinetics (bottom). Fifty millisecond continuous stimulus, effectively stimulates all neuron types (***A***), whereas a 300-ms-long pulsed stimulus (pulse repetition frequency (PRF) 100 Hz and duty-cycle 5%) causes only the LTS neuron to tonically fire a volley of APs (***B***). This selective LTS excitation is mediated through the elevation of the T-type calcium channels' S-gates open probability during the US off times (right), which elevates these channels' conductance and consequently amplifies the charge accumulation process that occurs during US's-on periods. ***C***–***E***, Threshold intensity versus duration required to generate a single AP using constant duty-cycle (PRF, 100 Hz). The excitation thresholds for the RS and FS neurons at 5% duty-cycle are >3.5 orders of magnitude higher than for the LTS neuron (***E***), decreasing rapidly to ∼2× at 50% duty-cycle (***C***).

We first studied the fundamental response of the three NICE-type models of cortical neurons to CW and low duty-cycle (5%) US stimulation, as used by Yoo et al. (2011a) to respectively excite or suppress cortical activity (Fig. 2; 0.69 MHz, acoustic pressure amplitude 320 kPa, intensity 3.3 W/cm^2^). During CW excitation, the US-frequency-driven oscillations of the NICE model neurons' intramembrane space lead to strongly hyperpolarized oscillations of the membrane potential, and the generation of a delayed AP train (after 10–25 ms; Fig. 2*A*); this excitation process occurs as a result of a charge accumulation mechanism described previously for the RS-NICE model (Plaksin et al., 2014). In contrast to this fairly uniform response of the three neuron types to CW excitation, their responses to low duty-cycle excitation is highly divergent (Fig. 2*B*), and only the LTS-type neuron is effectively excited by this excitation mode and tonically fires a volley of APs. Pulsed excitation with a low 5% duty-cycle and with varying US durations and frequencies, has excitation thresholds for LTS neuron that are upward of three orders of magnitude lower than those for FS and RS neurons; this ratio decreased rapidly as the duty-cycle increased, down to comparable thresholds at 50% duty-cycle (Fig. 2*C*–*E*). These excitation thresholds for the three cell types varied essentially independently of the excitation duration, and had only a weak dependence on US frequency, varying by <10% between 0.2–1 MHz (data not shown).

**Figure 3.**
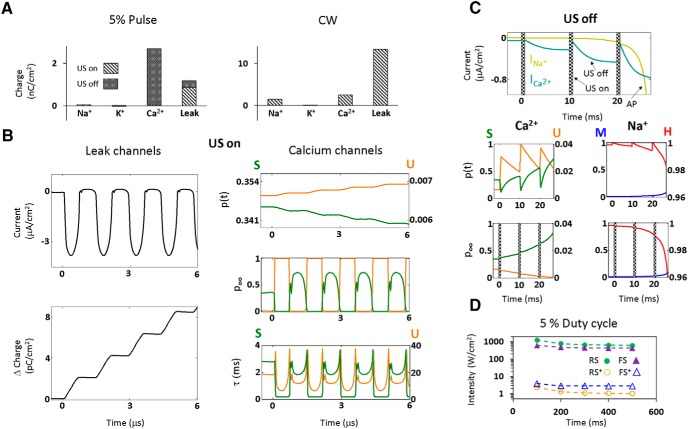
Detailed US response of LTS neurons (*f*=0.69 MHz). ***A***, The contribution of each channel type to the accumulated membrane charge during 10 ms of CW versus a short-pulsed US stimulus (5% duty-cycle, PRF=100Hz): leak channels have the biggest contribution during the US-on period, whereas the T-type calcium channels dominate the US-off period. ***B***, Leak and calcium channels' dynamical response to the first few US cycles (1.3 W/cm^2^); the hyperpolarized phase drives negative leak currents that insert positive charge into the cell, while rapidly suppressing the calcium conductance due to the changes in S- and U-type gates open probability *p*(*t*), through dynamical perturbations of the steady state probability (*p*_∞_), and the gates' time constants (τ). ***C***, T-type calcium versus sodium channels' dynamical responses during sparse stimulation (5% duty-cycle, 1.3 W/cm^2^); the comparison highlights the dramatic changes during the US breaks in the calcium currents, open probability *p*(*t*) and the steady-state open probability (*p*_∞_) of the S- and U-type gates, whereas the Na^+^ gates are mostly dormant prior to action potential initiation (arrow). ***D***, The pulsed US excitation thresholds of native RS and FS neurons versus following the chimeric addition of T-type calcium channels (RS+ and FS+).

What is the detailed biophysical basis underlying this selective AP generation by low duty-cycle US in the cortical LTS neurons? During US-on periods, the induced displacement currents cause a rapidly fluctuating hyperpolarized membrane potentials (Fig. 1*B*) that quickly suppresses the voltage-gated channels, whereas the non-voltage-dependent (leak) ion channels accumulate charge (Fig. 3*A*, left). Examining this process in high temporal detail (Fig. 3*B*), the asymmetric hyperpolarization is seen driving negative leak currents and charge accumulation while acting to close voltage-gated activation gates and to open the inactivation gates. The main difference between the cell types occurs during the US-breaks: the LTS neuron's T-type channels are the only VGCs that continue the charge accumulation process during these extended durations (Fig. 3*A*, left, *C*, top), due to intrinsic features that enhance their conductance during the US-off periods. Specifically, the contrast between the relatively slow recovery time of U-type gates (τ≈15 ms) versus fast recovery time (τ≈2 ms) of the S-type gates augments the channels conductance by allowing activation to recover relatively quickly (Fig. 3*C*, left) and net charge accumulation (eg, compare to the Na^+^ currents, which are negligible prior to AP initiation; Fig. 3*C*, right).

Finally, to test whether the T-type channels’ impact on low duty-cycle excitation is independent of the other specific biophysical properties of the LTS neuron model (eg, different AP thresholds parameters), we added these channels to RS and FS neuron models. This chimeric manipulation was found to create similar orders-of-magnitude differences in the neural thresholds and sensitivity to low duty-cycle excitation (Fig. 3*D*) through the same mechanisms.

**Figure 4.**
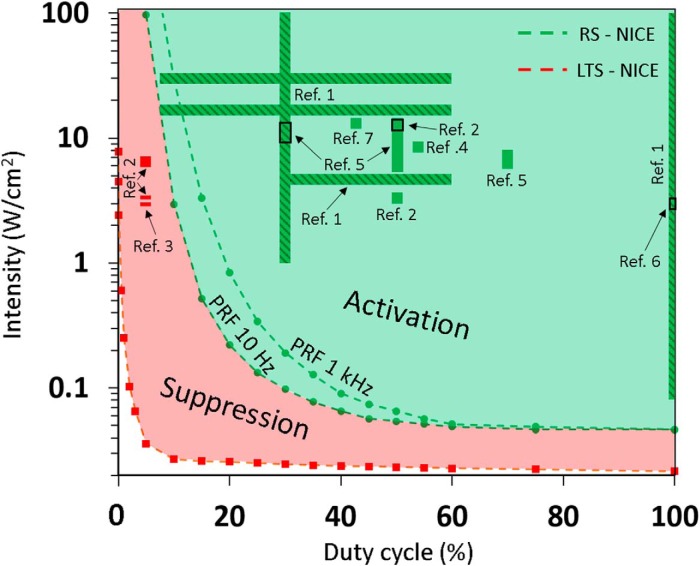
Phase plane diagram of single-neuron responses to varying US stimulation duty-cycle and intensity versus experimental cortical neuromodulation parameters. The phase diagram boundaries denote threshold intensities for US-mediated responses (frequency 0.69 MHz, duration 500 ms) from excitatory RS neurons (green dashed lines indicating 10 Hz and 1 kHz PRFs) and inhibitory LTS interneurons (red dashed lines, changes only slightly for different PRFs, not shown). These boundaries separate the phase diagram into regions where either the inhibitory LTS neurons are activated alone (red, “suppression zone”) or the RS and the LTS neurons are jointly activated leading to net network stimulation (green, “activation zone”). The superposed bars indicate the experimental parameter ranges used in seven published cortical ultrasonic neuromodulation studies, color-coded according to the mediated responses: Ref. 1 (King et al., 2013; bars with diagonal lines), Ref. 2 (Yoo et al., 2011a), Ref. 3 (Kim et al., 2015), Ref. 4 (Kim et al., 2012), Ref. 5 (Kim et al., 2014), Ref. 6 (King et al., 2014), and Ref. 7 (Tufail et al., 2011). The excitation parameters reported for King et al. (2013) were those that caused stimulation success rates significantly higher than their noise floor (∼20%), with low-frequency CW intensities corrected for the expected formation of standing waves (Plaksin et al., 2014).

Because the excitation thresholds are nearly independent of US frequency and duration, we next analyzed the joint dependence of the LTS and RS neurons on both duty-cycle and intensity, at a constant frequency and duration (0.69 MHz and 500 ms; Fig. 4). This analysis lead to a two-dimensional phase diagram that naturally separates into two activation domains where either LTS neurons are activated alone (at low duty-cycles) or both neuron types are jointly activated (at high duty-cycles). Our basic “first order” expectation (examined in detail in the next section) is that selectively exciting LTS inhibitory neurons will lead to network suppression, whereas jointly activating all neuron types with generally similar rates (where >75% are excitatory; Markram et al., 2004) will lead to net network stimulation. Next, we examined how these model-based predictions fit empirical data by marking on the parametric phase diagram the empirically-reported stimulation parameter ranges used in nearly all cortical ultrasonic neuromodulation studies (color-coded according to whether the reported effect was excitatory or suppressive). Strikingly, the result of this comparison (Fig. 4) demonstrates an excellent qualitative match between the model-based phase diagram and essentially the entire range of empirical stimulation parameters used for ultrasonic excitation and suppression. The diagram also shows that a two-order-of-magnitude variation in pulse repetition frequency (PRF changes from 10 to 1000 Hz) has a relatively minor influence on the response thresholds; anecdotally, the lowest duty-cycle empirical activation conditions (leftmost borders for King et al., 2013 results, Fig. 4) correspond to an intermediate PRF of about 400 Hz, and are seen to be in a fairly good agreement with the expected thresholds.

### Prediction II: effect of ultrasound on cortical network activity reflects cell-type selective responses and is modulated by thalamic inputs’ strength

**Figure 5.**
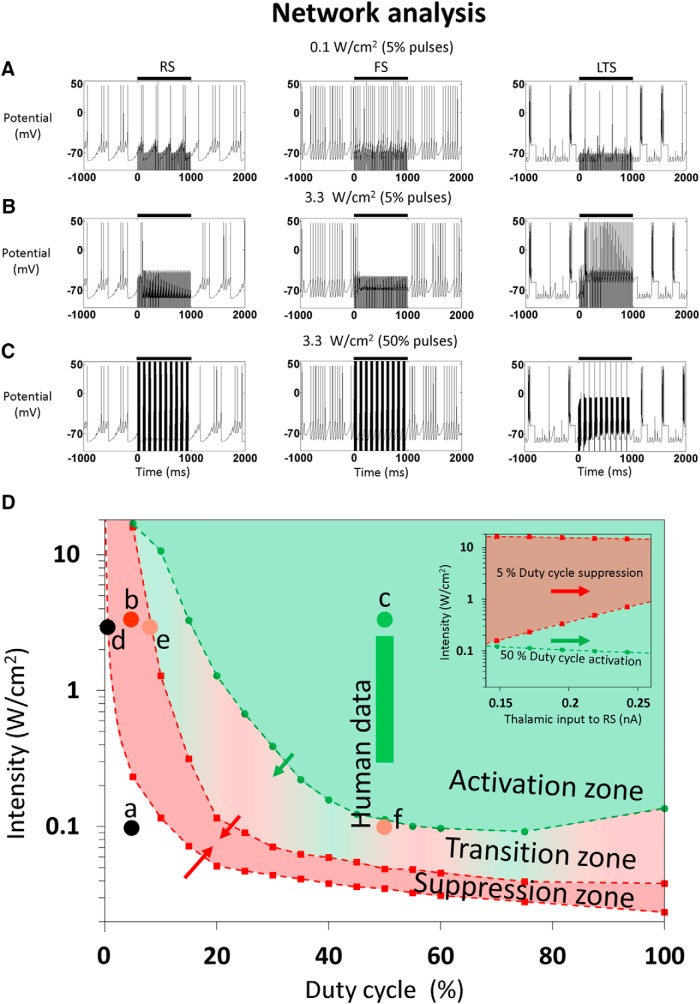
Simplified cortical NICE-network responses to different US waveforms and intensities. The US stimuli (US frequency and duration: 0.69 MHz and 1 s) are indicated by black bars (***A***–***C***). ***A***, For a stimulus duty-cycle of 5% and 0.1 W/cm^2^ intensity (PRF, 100 Hz) no significant response to US is observed. ***B***, Increasing the intensity to 3.3 W/cm^2^ causes FS and RS activity suppression due to strong LTS activation (∼40 Hz). ***C***, Increasing the duty-cycle to 50% (PRF, 10 Hz) leads to high frequency activation of the RS and FS neurons, unsuppressed by the weaker LTS firing (only at the beginning of each US pulse). ***D***, Phase plane diagram for the network responses to US with varying duty-cycle and intensity (PRF, 100 Hz). Marks a–c indicate the conditions of the respective simulations (matching the experimental observations of Yoo et al. (2011a) and marks d, e, indicate parameters from Kim et al. (2015) where the experimental responses were no longer suppressive. The vertical green bar represent human primary somatosensory cortex stimulation parameters used to evoke tactile sensations (Lee et al., 2015); f marks the only case where no response was observed. The green and red arrows and the inset depict the effect of increased thalamic input on the activation and suppression thresholds.

To examine how different US stimulation parameters affect cortical activity at the network level, we studied the responses of a simplified but physiologically plausible network population model to US pulses with varying parameters. The network contains the three cortical neuron population types studied above, where the RS and FS neurons receive thalamic inputs and all three NICE-neuron types are subjected to a 1-s-long US stimulus (Fig. 5, black bars). The model was adapted from Hayut et al. (2011) to studying the effect of US by moving from a rate-based network model to a H&H-based model connected by dynamic GABA_A_ and AMPA synapses, (Fig. 1*C*; see the Theoretical framework section for more information). When US stimulation is applied with a low 5% duty-cycle and intensity (0.1 W/cm^2^ or 56 kPa pressure amplitude, 100Hz PRF), no significant network response is observed (Fig. 5*A*), but at a higher intensity of 3.3 W/cm^2^ [320 kPa, used by Yoo et al. (2011a) and Kim et al. (2015) to obtain visual cortex suppression], the baseline activity of RS and FS populations was inhibited by the US-excitation of the LTS population, which responded at an elevated rate of ∼40 Hz (Fig. 5*B*). In contrast, using waveform parameters that were used to elicit motor cortical excitation: 50% duty-cycle and 10 Hz PRF, but having the same intensity (Yoo et al., 2011a), the RS and FS responses are dramatically increased by the extended US on times, suppressing the LTS responses and thus leading to overall excitation (Fig. 5*C*). These predictions from the network analysis are thus seen to be consistent with empirical findings; overall, the analysis predicts that the network's two-dimensional parametric phase diagram (Fig. 5*D*) naturally separates into a suppression domain, where LTS neurons are activated (at low duty-cycles), an activation domain, where the RS are activated, and a transition zone, where neither effect dominates. Interestingly, examination of experimentally tested neuromodulation parameters (Yoo et al., 2011a; Kim et al., 2015; Lee et al., 2015) with similar US intensities but varying duty-cycles or with the same 50% duty-cycle but varying US intensities, respectively, are found to have an excellent match with these predictions (Fig. 5*D*, points b–f and the green vertical bar). In these data either predominant suppression or stimulation are observed (points b, c, and the green bar; Yoo et al., 2011a; Lee et al., 2015) or a “transition” where neither clearly dominates (points d–f; Kim et al., 2015; Lee et al., 2015). Importantly, the zone borders are modulated by the strength of external (thalamic) input (Fig. 5*D*): increasing this input to the RS and FS cells lowers the threshold ultrasonic intensities required for RS stimulation and also shrinks the red suppression zone (Fig. 5*D*, inset). This shrinking effect is mediated indirectly: the stronger thalamic input drive synergistically compounds the US-induced excitation of the FS activity which acts to counteract the LTS's suppressive activity; this effect explains both the upward trend in the threshold drive required to excite LTS neurons and the (weaker) downward trend in the threshold required to overcome their effect (Fig. 5*D*, inset).

### Prediction III: thalamic neurons respond to low duty-cycle ultrasound, with exquisite sensitivity to waveform parameters

**Figure 6.**
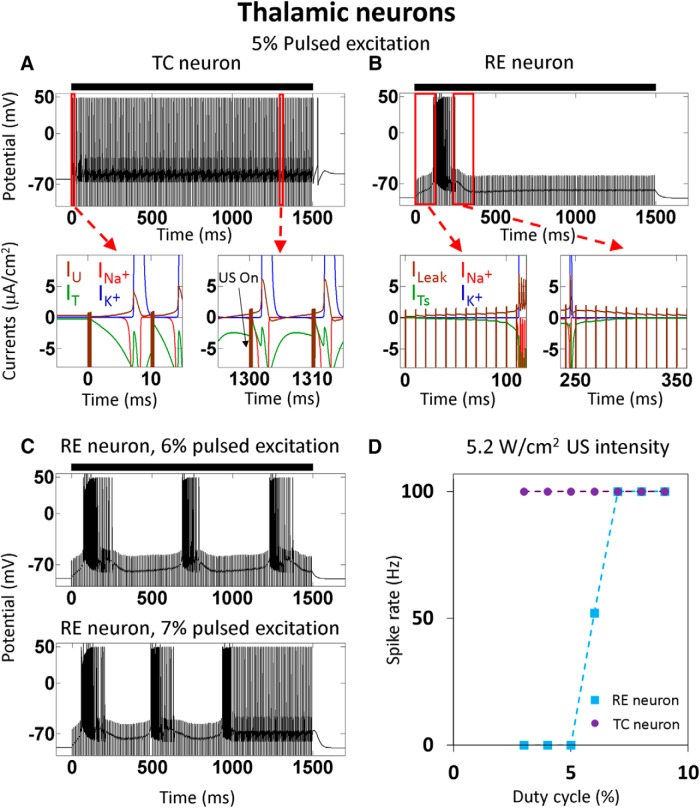
The response of thalamic NICE-TC and NICE-RE models to low duty-cycle US stimulation waveforms. The US stimuli (US intensity: 5.2 W/cm^2^; US frequency: 0.69 MHz; PRF, 100 Hz) are indicated by black bars (***A***–***C***). ***A***, ***B***, For a 1.5 s, 5% duty-cycle US stimulus, the TC cell fires a tonic 100 Hz volley of APs, whereas the RE cell fires only one volley and stops. Bottom, The currents' profiles of the segments marked in the top, where *I_U_* is the sum of *I_h_*, $I_{K_{L}^{+}}$, and *I_Leak_* currents (see complete channel composition in the Theoretical framework section). ***C***, Increasing the duty-cycle to 6% and 7% brings the RE neurons to fire periodical volleys and a constant volley of APs after two braked volleys, respectively. ***D***, The relation between the TC and RE neurons' spike rates and the US stimulation duty-cycle, calculated for the last 0.5 s period of the 1.5-s-long US stimulation.

Finally, we examined whether the characteristics of ultrasonic neuromodulation of thalamic circuits can be predicted by modeling the responses to ultrasound of two major thalamic neural cell types: thalamic RE and TC neurons, to the experimentally tested US pulse stimulation parameters (100 Hz pulses, 5% duty-cycle; Min et al., 2011; Yoo et al., 2011b; Yang et al., 2012). These neuron types also contain T-type calcium channels currents: I_T_ and I_Ts_ in the TC and RE neurons, respectively (Destexhe et al., 1998a; see Theoretical framework section). Both TC and RE neuron models were found to respond to low duty-cycle US stimulus (Fig. 6) with a common response mechanism that is related to the calcium channel dynamics (Fig. 6*A*,*B*, bottom), similarly to the one described above for the LTS cortical neurons (Figs. 2*B*, 3). However, whereas the TC neuron fires a tonic volley of APs almost immediately after the onset of the simulated ultrasonic stimulus, the RE neuron fires only one volley of APs before stopping. The TC neurons' calcium channels show stronger responses to both the hyperpolarized oscillations and the depolarized potentials, leading to stronger calcium currents in the US-off periods and therefore to a higher sensitivity to the US-pulse (Fig. 6, compare *A*, *B*, bottom left). In addition, the T-type calcium channels' S gates deactivation in the after-hyperpolarized state (following an AP) is relatively slow, and charge accumulation during this period repeatedly brings them to a state that is susceptible to re-excitation via brief bursts of applied US (Fig. 6*A*, bottom right). To explore the impact of these differential responses across a *range* of waveform parameters, we studied the models’ responses to waveforms with increasing duty-cycle, finding that even slight increases in the pulses' duty-cycle (from 5% to 7%; Fig. 6*B*,*C*) cause a dramatic shift in the US responses of the RE neuron type, eliminating its rapid adaptation to the US stimulus and pushing the neuron to a tonic firing regime; in contrast, the TC responses are not modified by these subtle changes (Fig. 6*D*). We also note that the T-type calcium channels in both of these thalamic neuron types have a much higher maximal conductance than in the cortical LTS neuron model (Table 1), leading to a generally higher level of excitability.

## Discussion

We theoretically studied the effect of this new mode of biomechanical-biophysical interaction between US and neurons with the goal of understanding the observed suppression of cortical neurons by low duty-cycle US. Such an understanding may guide the development of both therapeutic applications, such as seizure suppression (Min et al., 2011) and a myriad of research applications. The fundamental charge accumulation mechanism resulting from the imbalance of ionic currents during hyperpolarizing oscillations, which we have previously shown to underlie the generation of APs in cortical pyramidal (RS) NICE neurons (Plaksin et al., 2014), was found here to behave fundamentally differently in LTS neurons under low duty-cycle pulsed excitation. In these neurons, the overall suppression of all voltage-gated channels during the US-on periods (Fig. 2*B*) is accompanied by partial opening of the T-type calcium channels' U-gates; coupled with US-induced charge accumulation, this results in opening the calcium channels' S-gates, whose increased conductance effectively boosts membrane depolarization during the prolonged US-off periods (Fig. 3*A*, left, *C*, top) and leads to effective, cell-type selective excitation (Fig. 2*B*, bottom). Analysis of chimeric models of RS and FS neurons where the T-type channels were added further demonstrates their crucial role in this selective excitation (Fig. 3*D*).

**Figure 7.**
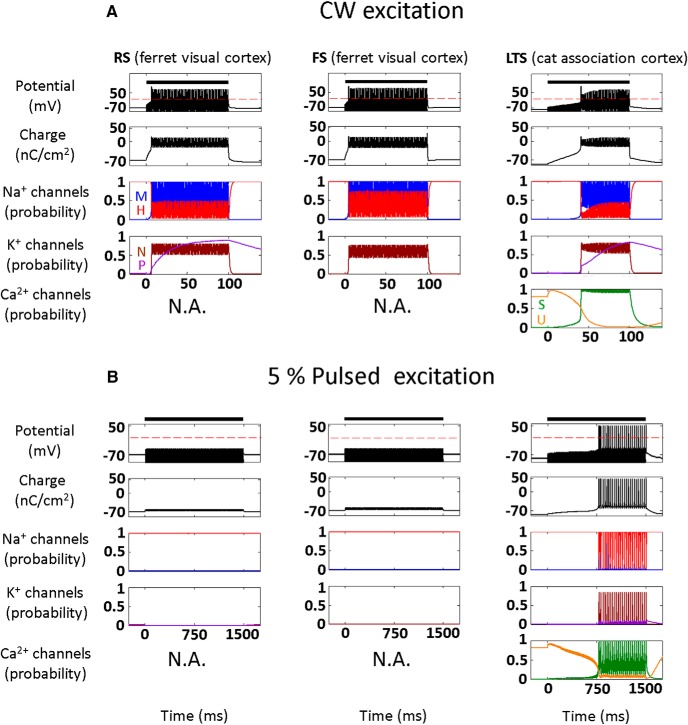
Effect on cortical NICE-neuron models from different mammalian species (Pospischil et al., 2008) of continuous and 5% duty-cycle pulsed US stimuli (US intensity: 3.3 W/cm^2^; US frequency: 0.69 MHz; PRF, 100 Hz, indicated by bars). US stimulus effects on membrane potential, charge and channels kinetics for cortical neurons of two different mammals (RS and FS, ferret visual cortex; LTS, cat association cortex). The panel organization and responses were similar to those described in Figure 2 and are explained by the very same underlying mechanisms. For continuous stimuli (***A***; 100 ms duration*)* there isn't a major difference between the responses of the different neuron types, except for a delay in the LTS neuron firing due to low leaky channels' conductances that cause slower charge accumulation. For pulsed stimuli (***B***; 1500 ms duration*)*, only the LTS neuron responded.

**Figure 8.**
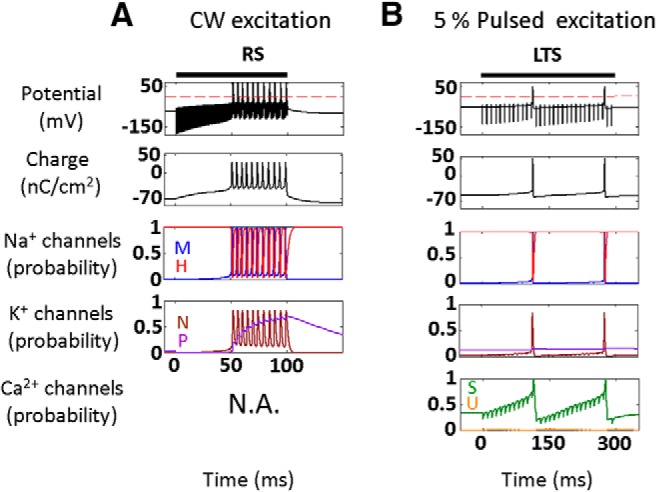
Effect of partial sonophore membrane area coverage during continuous and 5% duty-cycle pulsed US stimuli (US intensity: 3.3 W/cm^2^; US frequency: 0.69 MHz; PRF, 100 Hz, indicated by bars) on cortical RS (***A***) and LTS-NICE (***B***) neuron models, respectively. Partial coverage (here 75%) reduces the membrane potential oscillations down to a narrower range (>−150mV). Although the potential oscillations were more limited, the neurons' response to continuous and pulsed stimulation is still evident. Membrane capacitance was calculated as a weighted mean of the resting and dynamic capacitances: $C_{m}=f_{s}C_{m\_s}(t)+(1-f_{s})C_{m_{0}}$ , where *fs* is the active area fraction.

**Figure 9.**
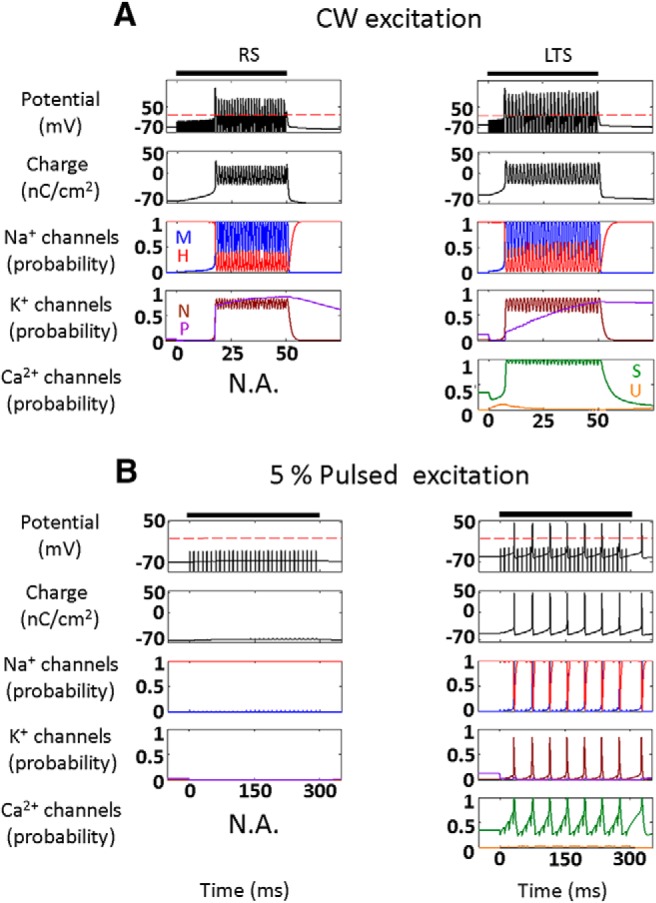
Effect of purely sinusoidal capacitive drive on cortical RS and LTS-neuron models in continuous (***A***) and 5% duty-cycle (***B***) stimulation modes ($C_{m}=C_{m_{0}}+C_{Amp}\sin(2\pi ft)$ , *C_Amp_*≈0.8 μF/cm^2^, *f*=0.69 MHz; PRF, 100 Hz, indicated by bars). $C_{m_{0}}$ is the resting membrane capacitance. Although the sinusoidal and the intramembrane cavitation theory-based capacitance variations are fundamentally different, the basic qualitative neural responses remain the same. The *C*_Amp_ was determined when 80% decline in the membrane capacitance (Fig. 1B; *f*=0.69 MHz and intensity 3.3 W/cm^2^) was taken into account.

The model-based predictions were found to provide a *post hoc* justification and understanding for the results of a significant body of experimental studies (Fig. 4), where parameter regimes naturally separated into suppression versus activation domains according to the pulse train’s duty-cycle, essentially independently of other stimulation parameters. This separation in terms of cell-type-selective interactions is, to the best of our knowledge, completely novel and was also found to carry over to the network level (Fig. 5). Here, additional interesting issues were observed including modulation of the suppression and stimulation thresholds by the thalamic input level (Fig. 5*D*) and the emergence of a transition zone between the suppression and excitation domains, where joint stimulation of multiple cell types results in a balanced state where none of the effects dominate. The network model's predictions have excellent agreement with very recently reported experimental results in rats (Kim et al., 2015), as well as in human primary somatosensory cortex (Lee et al., 2015), where artificial tactile sensations were reportedly evoked in all but one subject (Fig. 5*D*). Additionally, we note that these network-level observations could also explain several other effects that were associated with US stimulation, for example, it predicts a dependence of the US excitation parameters on the doses and type of anesthesia used, which is known to directly affect the level of thalamic activity (Alkire and Miller, 2005), moreover, it predicts that an extended ultrasonic suppression regimen will strongly drive LTS neurons and can lead to post-tetanic potentiation of their synapses with plasticity effects that can last for minutes after the stimulation ends (Storozhuk et al., 2005; Yoo et al., 2011a). Finally, we note that the network analysis predicts the existence of an optimum duty-cycle where the activation threshold is minimal (∼70%; Fig. 5*D*); this convex behavior, first predicted by Plaksin et al. (2014), reflects a tradeoff in RS and FS neurons between charge accumulation during US-on and the ability to discharge during US-off periods, and was recently experimentally validated by Kim et al. (2014). It is important to note that the model apparently has a very strong predictive power despite the multiple simplifying assumptions that our theoretical approach invokes regarding the biomechanics and biophysics of membranes, and ignoring the morphological complexity, which exists at both the single neuron and the network levels. This robustness could potentially indicate that the presented model already captures the essential features of the underlying biophysics, a conclusion supported by early analyses using NEURON models of detailed cortical NICE-networks and detailed analyses showing negligible accessory effects from radiation pressure (Prieto et al., 2013; M. Plaksin, S. Shoham, and E. Kimmel, unpublished observations). Moreover, inspecting the responses of NICE models with different sets of parameters adapted from cortical neuron models for ferrets and cats (Fig. 7), shows an insensitivity of the basic model-based US-induced behavior, and suggests that ultrasonic neuromodulation and its underlying mechanisms may be fairly universal among different mammalian species. In addition, NICE US-induced responses are robust to partial sonophore membrane coverage and the associated reduced membrane potential oscillation range (Fig. 8), nor do they strictly depend on the BLS biomechanics: the same qualitative results can be obtained by an arbitrary source of sinusoidal membrane capacitance variations (Fig. 9).

In comparison to the relatively smooth parameter dependence observed in the cortical simulations, it appears that key thalamic neurons display an even stronger cell-type-selective dependence on stimulation parameters. Interestingly, activity volleys in thalamic RE neurons play a key role in the formation of certain types of epileptic seizures (Slaght et al., 2002; Lee et al., 2014). The observed super-sensitivity of the NICE-RE model to the pulsed duty-cycle parameter could thus potentially underlie a predicted transition from suppression to seizures during very mild corresponding increases in the duty-cycle of US applied to the thalamic structures (from 5% to 7%), and perhaps explain why only 5% has been experimentally reported (Min et al., 2011; Yoo et al., 2011b; Yang et al., 2012).

In summary, we have demonstrated that the NICE framework, based on analyzing the expected effects of intramembrane cavitation on multiple cell types using well characterized, Hodgkin-Huxley type canonical models of membrane biophysics, not only agrees with, but also sheds new light on a large array US neuromodulation experimental results. Importantly, our results show that waveform modulation can potentially be used to selectively drive cell-type-selective responses leading to suppression. Achieving cell-type-selective stimulation is considered one of the crowning achievements of optogenetics, and it is thus fascinating to consider that this capability can also be translated and further refined to this noninvasive neuromodulation modality and potentially easily applied even to human subjects. While this study explored several major neural cell types of the neocortex and the thalamus, future studies could further explore the responses of other neuron types in additional brain regions, to achieve a more complete selective ultrasonic stimulation ability. In fact, the T-type voltage gated calcium channels are expressed not only in the neocortex and the thalamus, but also in hippocampal pyramidal neurons (Nikonenko et al., 2005), putatively explaining the ability to stimulate the hippocampus neurons by low duty-cycles and very low US intensities (Tyler et al., 2008; Tufail et al., 2010). Given the wide design space for acoustic excitation waveforms and strategies, these results highlight the potential of this model-based approach to optimized design for various brain regions and target applications.

## Synthesis

The decision was a result of the Reviewing Editor Marlene Bartos and the peer reviewers coming together and discussing their recommendations until a consensus was reached. A fact-based synthesis statement explaining their decision and outlining what is needed to prepare a revision is listed below.

Synthesis Statement for Author (Required):

All reviewers agreed that the manuscript in its current form does not give much insight into the mechanism how channels are differentially modulated. They are not convinced that the channel model the authors were using is biologically realistic under the conditions of ultrasound stimulation, not because it hasn't been fitted to experimental data, but because there is a discontinuity in the equations. The authors should either include an analysis leading to the conceptual understanding of differential ion channel activation and/or provide further experimental testing/evidence on the channel activation.

Reviewer 1:

The authors use a recently published model of the effects of ultrasound stimulation on (charged) membranes and apply it simplified cortical and thalamical neuron models with detailed Hodgkin-Huxley-type ion channel dynamics. They find that a particular channel, the T-type ion channel, is sensitive to ultrasound stimulation and changes its dynamics in a way that it tends to open after US stimulation. This tendency increases linearly with the time of stimulation and is relatively long-lasting, such that it allows stimulating these channels with a pulsed ultrasound, whereas other channels do not show this effect. They further state that this channel is mostly found in inhibitory neurons, opening a possibility to selectively stimulate them using pulsed ultrasound and match these results to a number of experimental findings. They proceed to analyze the implications on simulated networks of neurons and find that their results are robust to network effects.

I believe that this work has a potential to significantly advance our understanding of ultrasound stimulation of neurons, providing a mechanistic framework and making very specific predictions, which will allow experimenters to specifically target T-type ion channels to test this theory.

However, I also think that there is a lot that can be done to improve the presentation of the suspected underlying mechanisms that lead to this effect. This has been addressed somewhere hidden in Prediction 3:

"The TC neurons' calcium channels show stronger sensitivity to depolarized potentials, leading to stronger calcium currents in the US-off periods and therefore to a higher sensitivity to the US-pulse (comparing Fig. 5A and Fig. 5B, bottom left). In addition, the T-type calcium channels' S gates deactivation in the after-hyperpolarized state (following an AP) is relatively slow, and charge accumulation during this period repeatedly brings them to a state that is susceptible to re-excitation via brief bursts of applied US (Fig. 5A, bottom right)."

I think that the authors should elaborate more on that and specifically elucidate whether the effect can be attributed to an asymmetry in voltage dependence (as I suspect is the case for other ion channels) or an asymmetry in gating timescales (as hinted in that quotation). When looking at the equations, I found that there is indeed such an asymmetry in timescales for the U-type gate when V_x is set to -7 mV. But this also involves a discontinuous voltage dependency of that gate (\tau_u jumps from 300 to 516 at -80 mV), and I would like to see this effect using a smooth voltage dependency of \tau_u. It has been done for Figure 6 (using different channel parameters), but I'm not convinced that this is the same effect as in Figure 2.

Maybe the contributions of these two asymmetries (or maybe there is an entirely different cause?) could be studied separately in a toy model.

Another potentially relevant point is whether these neurons show Type I, Type II or Type II* behaviour as this influences the minimum current required to elicit action potentials and may therefore have an impact on the response at different stimulation strengths. In this context (and also to look at mechanisms) the following paper may be of interest:

"Ion channel degeneracy enables robust and tunable neuronal firing rates PNAS 2015 112 (38) E5361-E5370; doi:10.1073/pnas.1516400112"

Minor comments / suggestions:

Prediction 2:

There is a reference to an online Model which I haven't found.

Figure 1:

It would be nice to transform the network diagram into a more standard representation, both to include relevant information about the connectivity and making it more readable. Those two references may help.

Ehrlich M, Schuffny R. Neural Schematics as a unified formal graphical representation of large-scale Neural Network Structures. Frontiers in Neuroinformatics. 2013;7:22. doi:10.3389/fninf.2013.00022.

Nordlie, Eilen, Marc-Oliver Gewaltig, and Hans Ekkehard Plesser. "Towards reproducible descriptions of neuronal network models." PLoS Comput. Biol 5.8 (2009): e1000456.

Figure 4A:

It looks like LTS neurons do stop bursting and do not show lasting depolarizations during the bursts.

General:

Why are you using 690 kHz? In your last paper (Phys Rev X, Intramembrane Cavitation as a Predictive Bio-Piezoelectric Mechanism for

Ultrasonic Brain Stimulation), I found a comparison to King et al. between 250-600 kHz

How dependent are your results on the BLS theory? As this is a very new theory, there may still be modifications due to new experimental results in the future, and it would be of interest to identify the necessary components that makes the simulations in this paper work (i.e. would it be sufficient to replace that model with a high frequency oscillation in membrane voltage to obtain the main effects shown in this paper, or are there other essential components?)

It would be nice to actually see the response of I_h and I_M channels to US stimulation as they seem to change their gating properties for hyperpolarization.

The authors shouldn't reveal their identity in a double blind review process.

Reviewer 2:

This manuscript represents a HH model of the effects of ultrasound on network activity in the brain. The authors propose that through hyperpolarization of LTS neurons, ultrasound can enhance inhibition, because of rebound bursting, under certain conditions.

The proposed scheme is highly speculative and needs biological testing and support before publication. The results of manipulating certain cell classes will be very complex in the awake, interacting brain and depend upon not only the state of the animal, but also the precise wiring diagram of the circuit manipulated and how it was manipulated. Without this biological data, the accuracy of the model is completely unknown.

The first figure does not lend much confidence, since the authors show highly unrealistic swings in capacitance and membrane potential. Demonstration in real neurons of the expected effects of realistic levels of ultrasound at the cell are required to supplement the model.

The level of biological data that the authors quote to support their model is rather crude in comparison to the level modeled.
